# Insights into the Motif Preference of APOBEC3 Enzymes

**DOI:** 10.1371/journal.pone.0087679

**Published:** 2014-01-31

**Authors:** Diako Ebrahimi, Hamid Alinejad-Rokny, Miles P. Davenport

**Affiliations:** Complex Systems in Biology Group, Centre for Vascular Research, Faculty of Medicine, University of New South Wales, Sydney, New South Wales, Australia; Institut Pasteur Korea, Republic of Korea

## Abstract

We used a multivariate data analysis approach to identify motifs associated with HIV hypermutation by different APOBEC3 enzymes. The analysis showed that APOBEC3G targets G mainly within GG, TG, TGG, GGG, TGGG and also GGGT. The G nucleotides flanked by a C at the 3′ end (in +1 and +2 positions) were indicated as disfavoured targets by APOBEC3G. The G nucleotides within GGGG were found to be targeted at a frequency much less than what is expected. We found that the infrequent G-to-A mutation within GGGG is not limited to the inaccessibility, to APOBEC3, of poly Gs in the central and 3′polypurine tracts (PPTs) which remain double stranded during the HIV reverse transcription. GGGG motifs outside the PPTs were also disfavoured. The motifs GGAG and GAGG were also found to be disfavoured targets for APOBEC3. The motif-dependent mutation of G within the HIV genome by members of the APOBEC3 family other than APOBEC3G was limited to GA→AA changes. The results did not show evidence of other types of context dependent G-to-A changes in the HIV genome.

## Introduction

For many years it was not clear why a proportion of HIV sequences obtained from infected individuals contained G-to-A mutation footprints. Malim *et al.* discovered in 2002 a protein that was responsible for this mutagenic action against many affected HIV sequences [1]. Further studies revealed that this protein belongs to a 7-membered family known as APOBEC3 (apolipoprotein B mRNA-editing enzyme, catalytic polypeptide-like 3) several of which are known to mutate the HIV genome [2], [3], [4]. These enzymes are packaged along with the HIV RNA into nascent virions and upon release into a new cell they mutate C to U on the minus strand of HIV. This change is then observed as a G to A replacement on the HIV positive strand. Mutation by APOBEC3 enzymes are sequence context dependent. APOBEC3G primarily changes G nucleotides that are flanked by a 5′ T and up to two 3′ G nucleotides (TG, TGG and TGGG) [5]. But the other members of this family (APOBEC3A, 3B, 3C, 3D, 3F and 3H) tend to mutate G within GA [2]. For APOBEC3F preference within higher motifs (e.g. TGA and TGAA) have also been reported [6]. Mono- to tetra-nucleotides are referred to as motifs in this paper.

**Figure 1:**
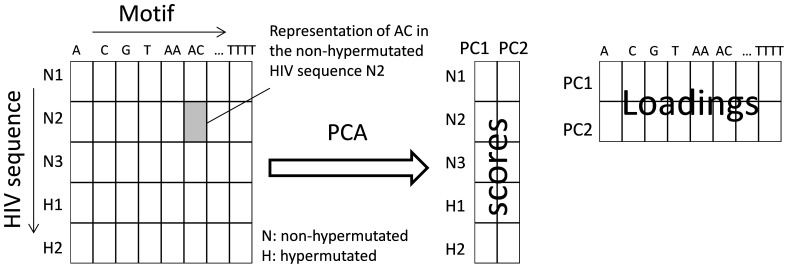
A schematic of principal component analysis applied to the motif representation data of HIV sequences.

Studies so far have investigated the motif preference of individual APOBEC3 enzymes on genome fragments and have mainly used *in vivo* or *ex vivo* experiments [6], [7], [8]. There is a limited number of studies in which full length *in vivo* hypermutated HIV genomes have been analysed to identify APOBEC3G and APOBEC3F target motifs [5], [9]. However in those studies the mutated motifs and the extent of hypermutation have been determined using an alignment to a constructed reference sequence that may not be the true ancestral sequence of the hypermutated sequences. Most importantly the *frequency* (relative abundance) of motifs, but not their true “*representations*” were compared to infer the motif preference of APOBEC3 enzymes [9]. Therefore in those analyses the expected mutability of motifs was ignored. To clarify this point let's consider two motifs, TGG and GGG, both containing the well-known APOBEC3G target dinucleotide GG. The trinucleotide motif GGG contains two overlapping GG dinucleotides, thus is more likely to be targeted by APOBEC3G compared to TGG with only one GG dinucleotide. Therefore GGG has a higher expected mutability compared to TGG. To be considered equally preferred target motifs, the reduction in the frequency of GGG in a given hypermutated sequence needs to be higher (nearly double) than that of TGG. This implies that simply considering the reduction in the *frequency* of a motif in a hypermutated sequence as evidence of motif preference can be misleading. We have previously shown that this issue can be overcome by quantifying motif “*representation*” using Markov models in which the frequency of constituents of each motif is taken into consideration [10], [11]. Representation is a measure of the abundance of motifs in the genome. The aim of this work is to identify the motif preference of APOBEC3 enzymes using an unbiased analysis of the hypermutation signatures that exist in the genome of affected HIV sequences. Additionally we aim to perform a comprehensive analysis of all motifs to investigate whether there is a novel mutational pattern and hierarchy in addition to already known GG→AG and GA→AA changes. The question we attempt to answer is “what is the motif preference of APOBEC3 enzymes when an unbiased multivariate analysis of full genome HIV sequences is performed?” Our approach is based on the fact that motifs such as GG and GGG which are preferentially targeted and mutated by APOBEC3 are less represented in the genome of hypermutated sequences compared to those of non-hypermutated sequences. The opposite is true for product motifs such as AG and AGG. Here we identify among all mono- to tetra-nucleotide motifs (340 in total; A, C, …, AA, AC, …, TTTT) those that are underrepresented (i.e. targeted motifs) or overrepresented (i.e. produced motifs) in the genome of hypermutated HIV sequences compared to those of normal (non-hypermutated) HIV sequences. For this purpose we analysed 2047 full genome HIV-1 sequences including 54 hypermutated sequences, belonging to the subtypes A1, B, C and the recombinant 01_AE, from naturally infected patients. We used a previously described method using Markov models [10], [11] to calculate the representation of motifs. To identify among motifs those associated with hypermutation (i.e. motifs whose representations increase or decrease in hypermutated sequences as a result of mutation by APOBEC3), it was necessary to employ an analysis approach that captures the association between HIV sequences and motifs. For each of 2047 HIV sequences we quantified the representation of all 340 mono- to tetra-nucleotide motifs. This data can be presented as a matrix (Fig 1) in which HIV sequences and motifs are rows (objects) and columns (variables), respectively. Each cell in this matrix is the representation of a particular motif in a particular HIV genome. Here each HIV sequence (row in the matrix) is defined using 340 motifs (columns in the matrix) and by the same token each motif is described using 2047 HIV sequences. An unbiased analysis of such a data matrix requires a multivariate approach such as principal component analysis (PCA) [12]. This method reveals the differences among the HIV sequences in terms of the representation of motifs. In other words, it discriminates between non-hypermutated and hypermutated HIV sequences. It also reveals the differences among motifs in terms of the HIV sequences. In other words it discriminates between motifs that do not change and those that change as a result of hypermutation. For the purpose of this study PCA is used to identify motifs that differ between non-hypermutated and hypermutated HIV sequences.

## Methods

### Quantification of motif representation using Markov models

The motifs targeted by APOBEC3 (e.g. TGG) are expected to be less abundant in the genome of hypermutated sequences compared to those in non-hypermutated sequences. Conversely, the motifs produced as a result of mutation (e.g. TAG, TAA) are expected to be more frequent in hypermutated genomes compared to those of non-hypermutated sequences. We have previously shown that Markov chains of probabilities can be used to quantify the “representation” of motifs and so to identify underrepresented and overrepresented motifs [10], [11]. In this method the expected probability (*P_exp_*) of each motif is calculated using the observed probabilities (*P_obs_*) of sub motifs. It is then compared to the observed probability of the motif using a ratio (*D*). An example is given in Eq. 1.



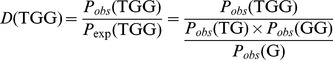
(Eq. 1)


In this method, the expected probability of the tri-nucleotide motif TGG is calculated using the observed probabilities of its dinucleotides (TG, GG) and mononucleotide G constituents. Therefore the calculated *D* ratio in eq. 1 quantifies the pure “representation” of TGG that is independent of the changes that may have occurred in TG, GG and G. We calculated the *D* ratio of all 340 motifs (mono- to tetra-nucleotide motifs) in 2047 full genome HIV sequences obtained from the Los Alamos National Laboratory (LANL) database. The sequences belonged to the subtypes A1 (n = 147), B (n = 1126), C (n = 514) and the recombinant 01_AE (n = 260) and contained both non-hypermutated and hypermutated sequences. The representation data matrix (2047×340) was used to perform principal component analysis.

### Principal Component Analysis (PCA)

Principal component analysis [12] is a multivariate data analysis technique that can be used to break down large data matrices into smaller and simplified matrices. It can be used as an exploratory analysis approach to investigate the grouping of objects (here HIV sequences), variables (here the motifs) and the association between objects and variables. A schematic of PCA applied to the motif representation data in the present study is given in Fig. 1. As indicated the representation data is a matrix. The data point in each cell of this matrix is the representation of one motif in one HIV sequence. This data matrix of size (HIV sequences × motifs) is decomposed into and described by two matrices, namely *scores* (HIV sequences × principal components) and *loadings* (principal components × motifs) as depicted in Fig 1. The scores and loadings matrices have reduced dimensions compared to the original data, thus are simpler to interpret. The scores matrix describes the relationship (similarity/dissimilarity) among HIV sequences in terms of latent variables (principal components) that are representative of the original variables (i.e. motifs). The loadings matrix contains information about the similarity/dissimilarity among motifs in terms of latent variables (principal components) that are representative of the original objects (i.e. HIV sequences).

In PCA, the principal components (PCs) are ordered based on their importance in terms of explaining the amount of variation in the data. The first PC captures the highest variance in the data. The second PC explains the second highest variation and so on. To investigate the relationship among the HIV sequences different columns of the scores matrix are plotted against one another. Similarly the groupings of motifs can be investigated by plotting different rows of the loadings matrix against one another. Here we analyse the data matrix of (2047 HIV-1×340 motifs) using PCA. As a pre-processing step the data was autoscaled. In other words each column of the matrix (i.e. each motif) was centralized by subtracting the data of the column from its average. The motif columns were then normalized by dividing the data in each column by its corresponding standard deviation.

### Accession numbers

The accession numbers of hypermutated sequences used in this study are: Subtype A1: EF165366; EF165365; AF484484; AF457091; AF457076; AF457071; AF457057; FJ388907. Subtype 01_AE: EF165361; AY945729; AY945723; AY945715; AY945714; AY358058; AY358055; AY358054; AY358053; GU201515; GU564226. Subtype B: EF165363; AY829213; AY037274; AY779556; AY781125; AY818643; AY818642; AY818641; AY531116; AY561241; FJ195087; FJ388922; FJ388897; JF689891; JF689888; JF689882; JF689881; JF689880; JF689878; JF689861; JF689858; JF689855; JN235961; EF178404. Subtype C: EF165360; EF165359; DQ275665; DQ164128; DQ164125; DQ164124; DQ164123; DQ056407; AY734561; AY734557; AY255828.

## Results

### Identification of subtypes

**Figure 2:**
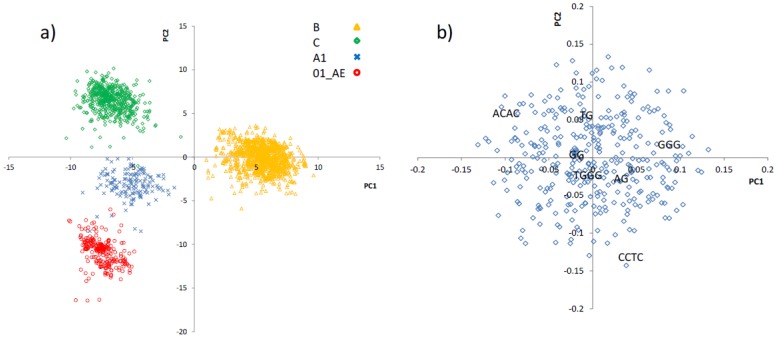
Principal component analysis of the motif representation data of HIV-1 sequences from subtypes/recombinant B, C, A1 and 01_AE. a) Scores plot (PC1 *vs*. PC2). Each point is a full genome HIV-1 sequence. PC1 differentiates between subtype B and other groups whereas PC2 discriminates among C, A1 and 01_AE. b) Loadings plot (PC1 *vs*. PC2). Each point is a motif. The broad distribution of motifs (identities of some of which are shown as examples) indicates that the difference among HIV groups is not due to a subset of motifs.

The major source of variation in the motif representation data comes from the subtype difference among the HIV-1 sequences. The first two principal components (PCs) capture this variation as depicted in the scores plot of PC1 versus PC2 in Fig 2a. In this picture four separate clusters each containing HIV-1 sequences from one subtype/recombinant is apparent. PC1 describes the difference between subtype B and other subtypes (C and A1) and the recombinant 01_AE. As displayed the scores of PC1 is positive for all subtype B HIV-1 sequences but this is negative for the other groups (C, A1, 01_AE). PC1 does not discriminate among these latter three. PC2, on the other hand, captures the information about the differences among C, A1 and 01_AE. These three groups appear as separated clusters along PC2.

To find out which motif is responsible for the observed differences among different HIV-1 subtypes/recombinant we explored the loadings plot (Fig. 2b). As displayed the motifs do not form clusters and appear uniformly distributed along PC1 and PC2. This indicates that the representations of almost all motifs are different between these four HIV-1 groups but similar within each subtype/recombinant. In other words the differences between these HIV families do not arise from a subclass of motifs.

### Identification of hypermutation by APOBEC3G

**Figure 3:**
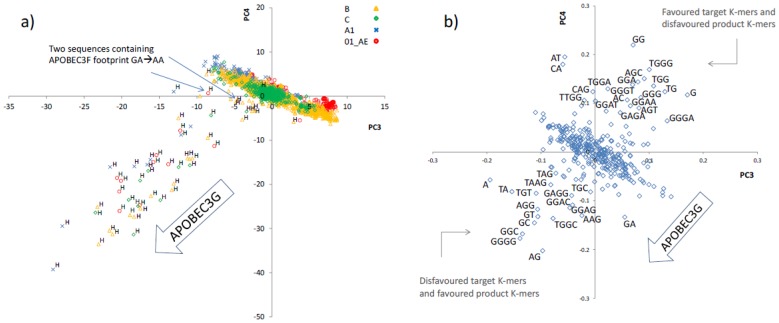
Principal component analysis of the motif representation data of HIV-1 sequences from subtypes/recombinant B, C, A1 and 01_AE. a) Scores plot (PC3 *vs*. PC4). Each point is an HIV-1 full genome sequence. Hypermutated sequences identified by the LANL database are indicated by “H” [11]. b) Loadings plot (PC3 *vs*. PC4). Each point is a motif. The motifs associated with hypermutation by APOBEC3G appear as outliers. Note that only two sequences (indicated by arrows) contain GA→AA footprints that is a hallmark of mutation by APOBEC3F and/or other similar APOBEC3 enzymes [11].

As indicated above the first and second principal components (PCs) mainly captured information about subtype differences among the HIV-1 sequences. To investigate whether there are PCs with information about hypermutation we explored all PCs up to 20. It was found that the third and fourth PCs contain information about footprint of mutation by APOBEC3G. That is, the 3^rd^ and 4^th^ PCs clearly separated out the sequences hypermutated by APOBEC3G. It is worth noting that the PCA model makes distinction between the sequences based on their subtype as well as their hypermutation status, without this information being used in the analysis. This analysis approach is referred to as unsupervised clustering [13]. Fig 3a depicts the distribution of HIV-1 sequences on the PC3-PC4 scores plot. In this figure the majority of HIV-1 sequences belonging to all different groups (subtype/recombinant) form a tight cluster; however several sequences appear as outliers that extend out of the main population in the same direction. These outlier sequences are all hypermutated by APOBEC3G (indicated by “H” on Fig. 3a) and have been identified as such by the Los Alamos National Laboratory (LANL) HIV database and also by our previous study [11]. This implies that these two PCs contain information about hypermutation by APOBEC3G.

The loadings plot of PC3-PC4 is shown in Fig. 3b. In this figure the majority of motifs appear as a cluster that has outliers extruded from it in two opposite directions. The loadings plot can be used to find the motifs (outlier motifs here) associated with hypermutation. For example GG, TG, TGG and TGGG that are known to be preferentially targeted by APOBEC3G appear at the top right hand side of Fig 3b. On the other hand AG, TAG and TAAG that are produced as a result of mutation by APOBEC3G appear in the bottom left hand side of this figure. Interestingly motifs such as GC, TGGC, GGGG which do not even contain an A nucleotide are found located within the product motifs. As will be explained later, these are disfavoured target motifs.

### Identification of hypermutation by other APOBEC3 members

**Figure 4:**
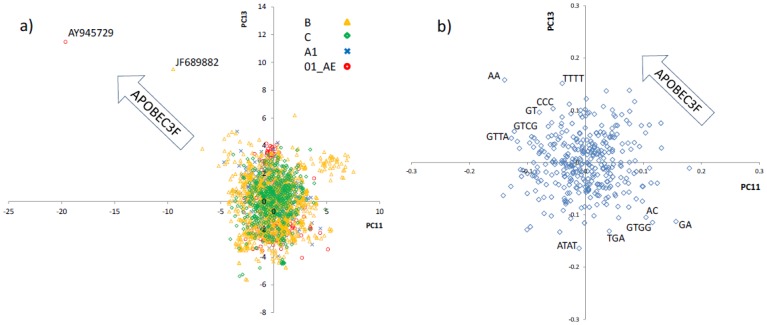
Principal component analysis of the motif representation data of HIV-1 sequences from subtypes/recombinant B, C, A1 and 01_AE. a) Scores plot (PC11 vs. PC13). Each point is an HIV-1 full genome sequence. The accession numbers of the two sequences that extend in the direction of mutation by APOBEC3F and/or other similar APOBEC3 members are shown near their points. b) Loadings plot (PC11 vs. PC13). Each point is a motif. The arrow shows the direction of GA→AA mutation by APOBEC3F and/or other similar APOBEC3 enzymes.

As will be explained in the discussion section, PC3 and PC4 only describe the differences between non-hypermutated HIV-1 sequences and those ones hypermutated by APOBEC3G. These PCs do not contain information about the footprint of other APOBEC3 enzymes. To identify the PCs that contain information about the mutation preference of other APOBEC3 members we searched through the remaining PCs. We looked for outlier motifs containing G and/or A, and being at the opposite ends of the loading plots. In other words we searched for outlier patterns similar to that shown in Fig 3b. We found that on the PC11-PC13 loadings plot (Fig 4b) the motifs GA and AA appear as outliers with their loading values being at the two opposite ends of the range. The inspection of PC11-PC13 scores plot (Fig 4a) revealed two outlier HIV sequences extending in the same direction as that of the motif AA shown in Fig 4b. This implies that the representation of AA increases in the genome of these two hypermutated sequences compared to that of other sequences including non-hypermutated and APOBEC3G-hypermutated sequences. Additionally, the motif GA is located in the opposite direction of AA, implying that GA is a target motif and that its representation decreases in these two hypermutated sequences compared to that of the rest of the HIV population. To further confirm that PC11-PC13 contains information about mutation by APOBEC3F and/or other similar APOBEC3 enzymes, that are characterized by a GA→AA signature, we analysed all 54 hypermutated sequences using the “hypermut 2” (http://www.hiv.lanl.gov/content/sequence/HYPERMUT/hypermut.html) as well as “hypersign” [11] programs. We found that only the two outlier HIV sequences identified on Fig 4a (accession numbers AY945729 and JF689882) contained a clear GA→AA mutation signature. In fact these sequences have been previously reported to contain GA→AA footprints [11].

Contrary to the induced mutations by APOBEC3G, hypermutation by APOBEC3F (and other APOBEC3 members) is not a major source of variation in the representation data. This is because very few sequences in the database (and so in the analysed data) have been affected by APOBEC3 members with a GA→AA mutation preference. Therefore the PCs that explain changes due to these enzymes are not expected to explain a large proportion of variation in this data. In other words the GA→AA hypermutation signature was not unexpected to be found on later PCs. Other principal components only described inter-sequence variations that were due to general HIV diversity, thus are not discussed here.

Importantly, our search for outlier G- and A-containing motif patterns did not reveal novel mutational signatures except those described above, i.e. GG→AG and GA→AA.

### Role of polypurine tracts in the preservation of the motif GGGG

**Figure 5:**
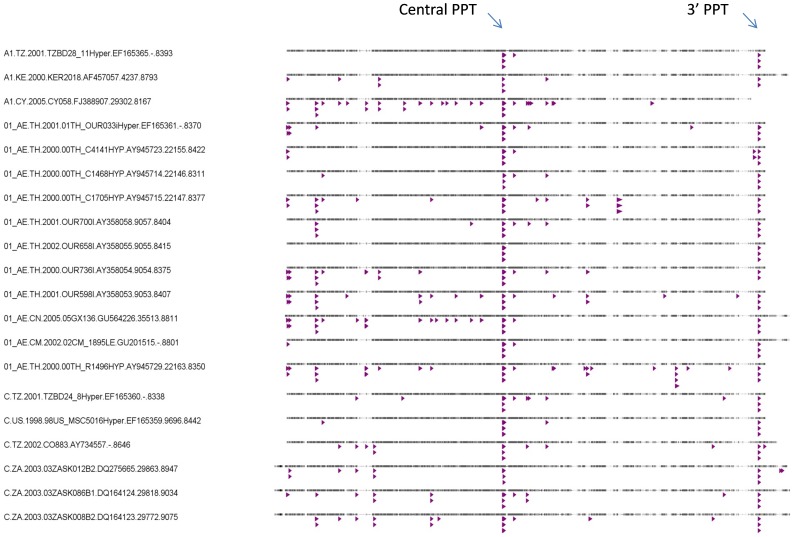
Conserved polypurine tracts shown in a representative alignment of 54 hypermutated HIV-1 sequences used in this study. Each line is a full genome hypermutated sequence within which the positions of GGGG motifs are shown by small triangles.

As will be explained later, the motif GGGG, which has been reported to be a highly favoured target motif of APOBEC3G, returned a high D value, thus appeared as a disfavoured motif in our analysis. We investigated the positional distribution of GGGG in the genome of hypermutated HIV sequences and found that GGGG is highly preserved at the central and 5′ polypurine tracts (PPTs). A representative alignment of hypermutated sequences with indicated GGGG motifs is shown in Fig 5.

**Figure 6:**
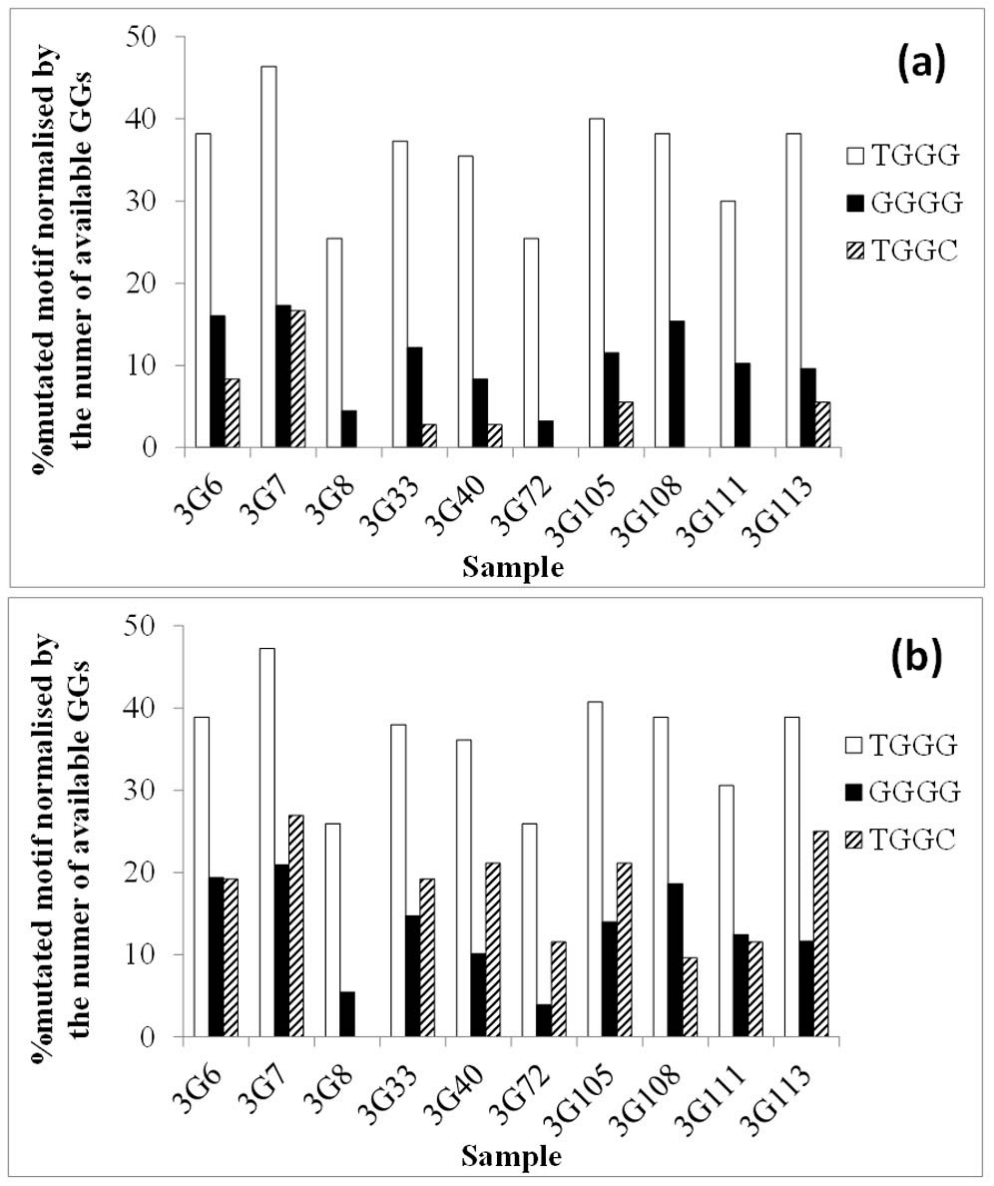
Percentage change of motifs within which G-to-A change(s) in the context of GG has occurred. The number of available GGs (i.e. mutability) has been used to normalize the results. a) Analysis of whole HIV genome; b) Analysis of the HIV genome without polypurine tracts. Hypermutated sequences (see reference [5] for details) are shown on the horizontal axes.

We asked the question “Is the preservation of GGGG motifs at PPTs the reason for high representation of GGGG in the HIV genome?” To answer this question we compared the percentage change of the motif GGGG (and two control motifs TGGG and TGGC) in the HIV genome with and without including the PPTs in the analysis. We used 10 full genome HIV sequences hypermutated in vitro by APOBEC3G [5] and calculated, in each sequence, the percent frequency of GGGG that changed to a different motif (compared to the non-hypermutated consensus sequence) as a result of G-to-A hypermutation within GG. For comparison we also calculated the same figure for TGGG (a known favoured motif) and TGGC (a known disfavoured motif). The results are presented in Figure 6a for the whole genome and in Fig 6b for the genome without the polypurine tracts. This analysis showed that GGGG is not a favoured target motif.

## Discussion

APOBEC3 proteins are important components of the immune system that inhibit infection by HIV and other similar viruses [14], [15], [16], [17]. They either act as mutagenic agents against HIV sequences or they control viral infection by other mutation-independent mechanisms [4], [18], [19], [20], [21]. APOBEC3G is known to mutate G within GG [8]. Other members of this family (e.g. APOBEC3F) prefer G within GA dinucleotides [2]. It has been shown for both APOBEC3G and APOBEC3F enzymes that the type of nucleotides flanking the target G from the 3′ as well as the 5′ end has an impact on the frequency of mutation of the targeted G. For APOBEC3G the presence of T at position -1 and G at positions +1 and +2 (relative to the target G at position zero) significantly increases the mutation frequency of the target G [5]. For APOBEC3F the presence of T at position -1 and A at positions +1 and +2 has been shown to have the same effect [9]. We have recently developed a bioinformatics method that identifies hypermutated sequences and discriminates between GG→AG and GA→AA footprints. In this method Markov conditional probabilities are used to quantify the representation of target (GG, GA) and product (AG, AA) dinucleotide motifs. We showed, using *in vivo* and *in vitro* [5] sequences, that sequences containing signatures of GG→AG, GA→AA or both can be uniquely identified [11]. Here we analyse all possible 340 motifs up to tetra-nucleotides in all non-hypermutated and hypermutated full genome HIV-1 sequences reported in the database to identify the motifs associated with hypermutation. In order to find this association we first quantified the representation of each motif using Markov models and then employed principal component analysis (PCA).

PCA returns a number of principal components describing major sources of variation in the data. In the motif representation data presented in this work there are potentially many sources of variation. Within these data there are variations arising from the subtype difference among the HIV sequences as well as those related to hypermutation by different enzymes. The PCs that capture the major variations are expected to be related to subtype differences. As displayed in the PC1-PC2 scores plot (Fig 2a) there are four clusters for the four different families of sequences (subtypes B, C, A1 and recombinant 01_AE). The major source of variation in the data that is captured by PC1 is due to the difference between subtype B sequences with the remaining sequence groups. Subtype B sequences have a positive score on PC1, but the remaining sequences have negative scores that are within the same range on this PC. This may suggest that subtype B is biologically more distinct compared to other subtypes. The sequences belonging to C, A1 and 01_AE are separated from one another on PC2. The almost complete separation of sequences by subtype/recombinant suggests that these HIV groups have different motif profiles, thus distinct biological origins. It also suggests that multivariate analysis of the motif representation data can be used as a diagnostic tool to identify the subtype of an unknown HIV-1 sequence.

On the PC1-PC2 loadings plot (Fig.2b) the motifs do not show multiple clusters with extreme loadings on the PC1 or PC2. This suggests that the differences among subtypes do not arise from individual or groups of motifs. Sequences with different subtypes/recombinants are different from one another because the representations of many of the motifs are different between subtypes/recombinants.

The hypermutated sequences studied here have “at least” two distinct footprints: GG→AG and/or GA→AA [10]. The former is attributed to APOBEC3G and the latter to the remaining members of the APOBEC3 family in particular to APOBEC3F. Thus in addition to subtype described above, we expect two sources of variations due to hypermutation. The majority of hypermutated sequences studied here have footprints of mutation by APOBEC3G therefore we expect the PCs describing the effect of APOBEC3G to be of greater importance (i.e. to have higher eigenvalues) compared to those describing the impact of other APOBEC3 members including APOBEC3F. As displayed in Fig 3a the scores plot of PC3-PC4 contains information about hypermutation by APOBEC3G but not APOBEC3F. Contrary to Fig 2a, in this figure different HIV-1 families (A1, B, C and 01_AE) appear as a single cluster but with outliers that extend out in the same direction implying that they are different from the main cluster due to the same mechanism that is subtype-independent. Here the cluster contains normal HIV-1 sequences and the outlier points are HIV-1 sequences hypermutated by APOBEC3G. The outlier sequences do not form a tight cluster because hypermutated sequences contain different levels of G-to-A mutations. This analysis suggests that multivariate motif analysis can be used to identify hypermutated sequences and the extent of hypermutation regardless of their subtype/recombinant type and importantly without the need for aligning to and comparing against a consensus sequence.

In the loadings plot of PC3-PC4 (Fig 3b) the motifs form a main cluster with outliers extruding from it in two opposite directions implying that they are associated with the same mechanism but acting in different directions. One group of outlier motifs extend in the same direction as those of hypermutated sequences shown in Fig 3a. In other words they extend in the direction of hypermutation by APOBEC3G. Therefore these are motifs that are overrepresented in the genome of hypermutated sequences compared to those of non-hypermutated sequences. The other group of outlier motifs extend in the opposite direction implying that they are underrepresented in the genome of hypermutated sequences compared to non-hypermutated sequences. The representation of the centrally clustered motifs do not differ between non-hypermutated and hypermutated sequences, therefore they do not extend in any direction.

Those motifs that are relatively overrepresented in the hypermutated sequences are either motifs that are produced as a result of mutation by APOBEC3G (i.e. favoured product motifs) or they are motifs that are not targeted by APOBEC3G (i.e. disfavoured target motif) but their sub motif constituents are favoured targets by APOBEC3G. Two examples are given below to clarify this.

The representation of AG (a favoured product dinucleotide) and GC (a disfavoured target dinucleotide) are shown in Equations 2 and 3.



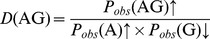
(Eq. 2)




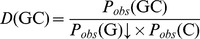
(Eq. 3)


The dinucleotide AG is a product of hypermutation (GG→AG) by APOBEC3G. Therefore its relative frequency increases in the hypermutated sequences (thus the arrow points up in Eq. 2). Hypermutation also increases the frequency of A and decreases that of G but the net result is an increased representation (*D*) for AG.

On the other hand the dinucleotide GC is not a product motif but is a disfavoured target motif by APOBEC3G. The frequencies of GC and C in the representation (*D*) equation (Eq. 3) do not change as a result of hypermutation but the frequency of G that is in the denominator decreases resulting in an increase in the representation of GC despite not being a product motif. Therefore the motifs overrepresented in the genome of hypermutated sequences compared to the non-hypermutated HIV sequences are either favoured product motifs or disfavoured target motifs. By the same token those motifs underrepresented in the genome of hypermutated sequences (those that extend in the opposite direction of the arrow printed with APOBEC3G in Fig 3) are either favoured target motifs or disfavoured product motifs.

The two motifs that appear at the two opposite and extreme ends are GG and AG (for simplicity only extreme outlier motifs are labelled in Fig. 3b). The high preference of APOBEC3G for GG and its replacement by AG that is indicated from Fig 3b is well known [5], [22]. Fig 3b also identifies other highly favoured targets by APOBEC3G such as TG, TGG, GGG and TGGG. The motifs that are produced by G-to-A mutation of these motifs are also identifiable from this plot. TA, TAG, AGG and TAAG appear relatively overrepresented in the hypermutated HIV sequences.

The motif GGGT also appears as a highly favoured target by this enzyme, implying that rows of three G nucleotides flanked by a T nucleotide at the either end are frequently targeted by APOBEC3G. The tetranucleotide GGGG not only does not appear as a target motif but also extends in the opposite direction, i.e. in the direction of product motifs. Given that this motif contains only G but not A, it cannot be a product motif. This implies that GGGG is a disfavoured target. In other words it is targeted at a frequency that is much less than what is expected from a motif that contains three overlapping GG targets. It is not clear why Gs within GGGG are relatively less favoured by APOBEC3G but it may be due to the formation of high order structures as a result of base pairing that renders Gs within GGGG inaccessible to APOBEC3G which mainly mutates G on single stranded DNA. It is known that the HIV genome remains double stranded at its two polypurine tracts (central and 3′ PPTs) during reverse transcription [22], [23], [24]. These two regions, despite having stretches of G nucleotides, are known to be immune from mutation by APOBEC3G whose substrate is single stranded DNA. The central polypurine tract (cPPT) of the HIV-1 genome contains two stretches of poly G (six Gs each) nucleotides separated by three nucleic acids. Additionally there is a stretch of four Gs about 9 nucleotides downstream of the second poly G. The 3′ PPT of the HIV-1 genome contains a single poly G (six Gs). The G nucleotides within these PPTs are expected to have remained intact in the genome of hypermutated HIV sequences, therefore have contributed to an elevated representation (*D* value) of the motif GGGG. To investigate this we first determined the positional distribution of GGGG in the genome of hypermutated HIV sequences. We found that in the hypermutated sequences, the poly Gs within PPTs have remained intact (see Fig 5).

To investigate whether it is the conservation of GGGG at PPTs that makes this motif appear as a disfavoured target in general, we compared the percentage mutation within this motif in the HIV genome with and without including the PPT regions. For this purpose we calculated the percentage of motifs within which one or more G-to-A change(s) in the context of GG (underlined G being the target) has occurred in 10 HIV sequences hypermutated *in vitro* by APOBEC3G [5]. The percentage of changes was normalized by the number of available GG dinucleotides within each motif. The results are presented in Figures 6a and 6b for the HIV genomes including and not including PPTs, respectively. As displayed in both cases the percentage of targeted GGGGs by APOBEC3G is closer to that of TGGC which is a disfavoured motif compared to that of TGGG which is a favoured target. This suggests that the infrequent targeting of GGGG by APOBEC3G is not due only to PPTs but instead is a general feature of this motif in the HIV genome.

The overrepresentation of other motifs that are not product motifs such as GC, GT, GGC and TGGC suggests that these motifs, despite having G, are usually disregarded by APOBEC3G. It is known that G nucleotides that are flanked by a C nucleotide at the +2 position (such as GGC and TGGC identified in Fig 3b) are not usually targeted by APOBEC3G [5], [22]. The high representation value of GC suggests that G nucleotides flanked by a C at +1 position are also disfavoured targets.

The higher representation of the 4-mer motifs GAGG and GGAG and the lower representation of their corresponding product motifs GAGA and GGAA in hypermutated sequences relative to non-hypermutated sequences suggest that GAGG and GGAG are also targeted less frequently than expected.

Our analysis also identified motifs associated with the mutation by other members of the APOBEC3 family such as APOBEC3F. In Fig. 4b the most preferred APOBEC3F target (GA) and its product (AA) appear as outliers of the rest of the motif population. Most importantly they appear in opposite directions implying the same mechanism acting in opposite directions in terms of the changes induced in the representation of these two motifs. In this PC plot no other motif appears to extend in either direction to the extent that was observed for APOBEC3G in Fig. 3b. Therefore the *in vivo* mutation of these two full genome HIV-1 sequences by APOBEC3F (and/or similar APOBEC3 members) has not left any evidence to suggest that there is a significant hierarchy of mutational preference beyond the dinucleotide GA by theses enzymes. However we note that in a previous study, using *in vitro* analysis of fragments of the HIV genome, the motif preference of APOBEC3F have been reported to go beyond dinucleotides [6]. This might suggest that other members of the APOEBC3 family are responsible for the GA→AA signature observed on these two genomes.

The direction of the extension of already known HIV-1 sequences with footprint of extensive GA→AA changes in Fig. 4 confirmed that these two PCs describe mutation by APOBEC3F and/or other members with a GA→AA mutation preference. This comprehensive motifs analysis also enabled us to conclude that the *in vivo* hypermutated HIV sequences do not contain any unknown mutational footprint beyond those of GG→AG (and its higher motif hierarchy) and GA→AA.

The dinucleotide GA which is the most targeted motif by APOBEC3F is also the second preferred target by APOBEC3G. The footprint of APOBEC3G is not only GG→AG but also includes GA→AA, though to a lesser extent. Therefore the principal components attributed to the action of APOBEC3G (see Fig 3), capture slight GA→AA changes, in addition to the main GG→AG changes. It is for this reason that the two sequences with mainly GA→AA signatures (attributed to the action of other APOBEC3 such as APOBEC3F; indicated by arrows in Fig 3a) appear slightly outside the cluster of non-hypermutated sequences, in nearly the same direction as those of APOBEC3G-hypermutated HIV. However on those principal components that describe exclusively GA→AA changes (i.e. PC11, PC13) it is only these two hypermutated sequences that appear as outliers. The rest of hypermutated sequences (i.e. APOBEC3G-hypermutated sequences) are grouped with non-hypermutated sequences on these PCs. The number of reported full genome *in vivo* hypermutated HIV sequences with GA→AA signatures is very limited. Therefore the target and product motifs of enzymes such as APOBEC3F are identified with less confidence compared to those of APOBEC3G.

## Conclusions

The analysis of motif representation data in a large population of full genome HIV-1 sequences using Markov models and multivariate data analysis revealed details of the mechanisms of mutation induced by individual APOBEC3 proteins. The highly favoured/disfavoured motifs and their products were identified in an unbiased approach and also the proposed method was shown to be useful for HIV subtyping.
